# Health Benefits of Air Quality Improvement: Empirical Research Based on Medical Insurance Reimbursement Data

**DOI:** 10.3389/fpubh.2022.855457

**Published:** 2022-03-03

**Authors:** Ding Li, Han Xiao, Shuang Ma, Jiangxue Zhang

**Affiliations:** ^1^School of Public Administration, Southwestern University of Finance and Economics, Chengdu, China; ^2^School of Economics, Southwestern University of Finance and Economics, Chengdu, China; ^3^School of Economics and Statistics, Guangzhou University, Guangzhou, China; ^4^Beijing Key Lab of Study on Sci-Tech Strategy for Urban Green Development, School of Economics and Resource Management, Beijing Normal University, Beijing, China

**Keywords:** air pollution, health benefits, medical insurance reimbursement data, induced demand, thermal inversion

## Abstract

Measuring the health benefits of air quality improvement is a new perspective for evaluating government investment in pollution control. Improving air quality can reduce the burden on medical insurance funds and patients themselves; however, patients with higher reimbursement rates are more affected by air quality changes. This study calculated health benefits using medical insurance reimbursement data from a sample city in China. The results show that for every 10 μg/m3 decrease in PM2.5, patients' average medical cost will decrease by CNY 1,699 (USD 263.6), and the loss of ordinary working and living time will decrease by 1.24 days. PM2.5 has a more significant impact on patients with chronic respiratory diseases and inpatients with circulatory diseases. Suppose the city's annual PM2.5 concentration drops to the national standard of 35 μg/m^3^. In that case, it will bring more than CNY 1.28 billion (USD 198 million) in health benefits, accounting for 18% of the city's annual investment in environmental protection.

## Introduction

Air pollution control is the focus of ecological development in many developing countries, and healthcare costs due to air pollution are receiving higher priority worldwide. Existing studies have constructed a well-established framework for research on air pollution and health outcomes, finding that air pollution is associated with higher infant mortality, morbidity, and healthcare expenditures (1–13).

However, since existing research has generally studied the relationship between poor air quality and health outcomes, evaluating “better air quality” is meaningful, especially for developing countries facing development transformation challenges (14). In 2013, the Chinese government issued an action plan for air pollution prevention and control^1^ that included systematic measures to combat air pollution. Existing evaluative studies have also found that over CNY 60 billion investments have resulted in significant improvements in air quality^2^ (15). Essentially, lower air pollution figures are meaningless to the government. Further studies are needed to evaluate the effectiveness of this investment in environmental public finance. Air quality improvement plays an essential role in enhancing productivity from a supply-side perspective (16); however, the health benefits need further study from a demand-side perspective. Public disclosure of environmental information has increased the population's overall health awareness and commitment, accompanied by increased proactive and defensive investments to address air pollution (21). Owing to consumption inertia, improvements in air quality will not change the established consumption habits of those who can defend themselves. However, for people who do not have sufficient self-defense capacity, the health costs resulting from air pollution will be high, which could cause forward health inequality between different people.

This study evaluated the benefits of environmental inputs from a new perspective. At the individual level, air quality improvements are mainly reflected in reduced healthcare costs, which are analyzed according to the morbidity of the two central air pollution-related diseases, namely respiratory and circulatory diseases. In addition, we introduce the concept of lost work hours based on Grossman's (22) health stock model; that is, air pollution may lead to a loss of wage income for patients due to the treatment of illnesses. Chen et al. (23) noted that workers facing air pollution might choose to forego income, highlighting the opportunity cost concept extending the traditional evaluation of health benefits. A link between the environment and the health sector is evident at the government level. Environmental inputs can lead to savings in health insurance spending, and establishing such a relationship will help the government improve the multiplier effect concerning financial inputs.

Estimation with two-step linear squares (2SLS) shows that for every 10 μg/m^3^ reduction in PM_2.5_, healthcare expenditures for patients with respiratory and circulatory diseases will decrease by 16%, and the visit duration in days will be reduced by 14%. These values remained robust after changing the instrument variables, adjusting the data structure, and accounting for the effects of population migration and air pollution warnings. Heterogeneity analysis found that air pollution has a more significant impact on the cost of visits for patients with chronic respiratory diseases and inpatients with circulatory diseases and a more substantial effect on the expenditure of drugs and treatment. Further research has found that improvements in air quality can alleviate both the burden of health insurance and individuals. This study found a moderating effect of health insurance reimbursement in the impact of air pollution on health care costs, where patients with higher reimbursement rates are subject to “induced demand” and will potentially face higher healthcare costs. We also find non-linearity, which shows a marginal health benefit increasing for PM_2.5_, under 90 μg/m^3^. In terms of economically significant measures, this study found that for every 10 μg/m^3^ reduction in PM_2.5_, the health benefits for respiratory and circulatory patients in the city would be CNY 546 million (USD 84.9 million). In contrast, a reduction in PM_2.5_, the annual average standard of 35 μg/m^3^ would result in annual health benefits of approximately CNY 1.278 billion (USD 198 million), including CNY 1.032 billion (USD 160 million) in health insurance expenditure savings.

The contributions of our study can be summarized as follows: First, we expanded the calculation range of health benefits for air quality improvement by introducing the concept of lost work hours. Specifically, we estimated the opportunity cost of treating illness by regressing the visit duration on air pollution. Second, we provided a more comprehensive measure of the health benefits of air quality improvement. We calculated the health benefits in the resident and government sectors based on the proportion of costs reimbursed by health insurance. Third, we estimated the causal relationship between air quality and medical health costs. We used a high frequency with extensive observation data from the medical insurance reimbursement system to capture the health cost of patients. Furthermore, we analyze this relationship using instrumental variables to solve the endogeneity.

This paper continues in the following manner. Section Literature Review presents the literature review; Section Theoretical Model is a refinement of the Grossman health stock model; Section Data and Variables is a basic description of the research data; Sections Empirical Methodology, Empirical Results, and Further Analysis present the methodology and results of the baseline empirical analysis and further analysis, respectively; and Section Conclusions provides concluding remarks.

## Literature Review

Research on how air pollution affects health falls into two perspectives: the impact of air pollution on people's health outcomes and the cost of defending against air pollution. Chen et al. (2) used total suspended particles (TSPs) as the measurement index of air pollution. They estimated the negative impact of air pollution on life expectancy by taking the heating difference between the north and south of the Huai River as a geographical regression discontinuity design. Janke (3) found that nitrogen dioxide (NO_2_) and ozone (O_3_) had a significant positive impact on children's respiratory emergency hospitalizations in England. Schlenker and Walker (7) measured the impact of carbon monoxide (CO) emissions caused by taxiing time of airplanes at the airport. They found that CO significantly increased the morbidity of residents within 10 km of the airport. Deschenes et al. (10) found that air pollution affects body weight through biological channels and causes overweight through behavioral channels, such as increasing calorie intake. Many other studies have focused on the relationship between air pollution and mortality (4–6, 9, 12). The above studies focused on the externalities of air pollution, which is conducive to a deeper understanding of the negative impact of air pollution on working and living.

Another perspective focuses on the cost of defending against air pollution. Existing studies have generally found that air pollution has contributed to increasing mask and air purifier sales (17–19) and increasing commercial insurance purchases (20). Deschênes et al. (24) and Deryugina et al.'s (9) examination of the relationship between air quality and health expenditure in the US healthcare market provided many references for our study; however, the three studies based on the Chinese situation are the most relevant to us. Barwick et al. (25) used China UnionPay credit card consumption data for the period 2013–2015 to study the impact of air pollution on morbidity costs and found a significant relationship between air pollution and health expenditures; they measured the decline in health or non-health consumption when the PM_2.5_ falls below the World Health Organization's (WHO) air quality standards as the morbidity cost of air pollution. Liao et al. (26), however, used data from the 2016 and 2018 China Family Panel Studies (CFPS) and found that PM_2.5_, which increases the cost of air pollution by affecting lifestyle (sleeping time and sedentary activities hours), will increase household health care expenditure and out-of-pocket hospital expenditure, and that the effect is more pronounced in younger age groups. Shen et al. (27) focused on the impact of industrial air pollution on health expenditure by region using province-level panel data for the period 2002–2015. They found that air pollution has a greater effect in central and eastern China and a non-linear impact in western China, with a negative impact below the threshold.

In the above studies, Barwick et al. (25) tracked credit card spending in hospitals, Liao et al. (26) utilized information from micro surveys to determine the nature of consumption, which can help researchers identify how air pollution contributes to health expenditure more clearly, both of which were studied from a consumer perspective. We analyzed the medical insurance reimbursement system from a provincial capital city in China for our research. There are some differences between our study and the above literature. First, our data facilitate more accurate identification of medical expenditure from the hospital perspective and allow us to avoid bias due to different payment methods or respondents' subjective judgments. Second, compared to consumer data, the data obtained from hospitals contain richer information about individual medical histories, enabling the study to be conducted in the appropriate pathological context. Furthermore, these data include the specific visit dates for all patients from January 2016 to December 2017; hence, the data better capture daily air quality trends by matching daily air pollution indicators, whereas the annual data of Liao et al. (26) and Shen et al. (27) used only the air quality for the entire year.

However, we should admit that the medical insurance system data still has inevitable shortages. Due to data source limitations, our data cover only 2 years, and it is difficult to prolong the period of data. However, our data might be the newest accessible data from the medical insurance system. As we know, the current data wave also allows us to control series time fixed effects such as year fixed effects, month fixed effects, and day of week fixed effects. Our study also faces the problem of lacking data from more cities. We believe that our research introduces a model to evaluate the health benefits of environmental improvement.

## Theoretical Model

Grossman (22) analyzed health needs and inputs in a utility maximization framework. This study serves as the basis for various theories (24, 25). Deschênes et al. (24) considered the marginal willingness to pay (MWTP) for air pollution in terms of patients' duration of illness due to the effects of air pollution. Barwick et al. (25) further considered the time of exposure to a polluted environment. They subdivided consumption into medical, online non-medical, and offline non-medical consumption. The latter two ultimately yield MWTP for air pollution by solving for the maximization of the utility of the health stock, subject to budget constraints. Based on the above studies, this paper discusses the boundaries of health benefits concerning the characteristics of empirical data.

In this study, MWTP is defined as the health benefits of air quality improvement, mainly for the following reasons. On the one hand, MWTP refers to the cost of optimal healthcare spending, which alleviates the negative impact of air pollution exposure in the model of Grossman (22). It can also be regarded as the opportunity cost of better health outcomes and better living progress with given air quality. This cost will decrease with improved air quality, which means that the health benefits of air quality are another side of the MWTP for air pollution.

On the other hand, the MWTP measures the subjective intention of expenditure in most situations; however, the health benefits can not only reflect the individual revenue of patients who go to the hospital to get better health outcomes with a proactive attitude contain the objective gains of government environment investment. Thus, the health benefits of air quality improvement have enriched the traditional concept of MWPT for addressing air pollution.

For the model, the first is the health stock equation, given that it consists of three components. *h*_0_ denotes the health stock of the patient's endowment, *m* denotes patients' medical expenditure to cope with air pollution, and medical spending can improve health status. *g*(*e*) is the health loss due to air pollution, and *e*(*a, m*) is the loss of working hours due to air pollution. The higher the pollution level, the more working hours lost; therefore, there is a positive relationship with the level of air pollution. The relationship between health expenditures and lost working hours is uncertain, but the direction of the coefficient does not influence the final result. It is certain that the more working hours lost (time spent on visits), the greater the overall health loss. Therefore, *g*(*e*) is an incremental function of *e*(*a, m*), as in the existing literature (25). The three components of the health stock are summed linearly in this study, as follows:


(1) $$\begin{array}{l} h=h_{0}+m-g\left(e\right) \end{array}$$

In the long run, total income consists of two components: non-wage income, *y*_0_, and wage income, *W*[*h*(*e*), *e*], where wage earnings are proportional to health status and inversely proportional to working hours lost, with a budget constraint, as in Equation 2:


(2) $$\begin{array}{l} y\left(h\right)=y_{0}+W\left(h\right)\geq \pi +pm+c \end{array}$$

π indicates that the insurance pays an annuity, *p* is the proportion of out-of-pocket payments, and *c* is non-health expenditure. As this study does not focus on consumption information other than medical expenditure, it treats *c* as exogenously given.

Final utility *U*(*h, c, e*) is affected by three components: health status, loss of working hours, and expenditure. Equation 5 was first obtained using the Lagrangian first-order derivation:


(3) $$\begin{array}{l} L=U\left(h,c,e\right)+\lambda \left[y\left(h\right)-\pi -pm-c\right] \end{array}$$

(4) $$\begin{array}{l} \frac{\partial L}{\partial m}=0 \end{array}$$

(5) $$\begin{array}{l} \lambda p=U_{e}e_{m}+\left(U_{h}g_{e}e_{m}+\lambda y_{h}\left[1-g_{e}e_{m}\right]\right) \end{array}$$

The MWTP for air pollution can be obtained using the implicit function theorem, as shown in Equation 6.


(6) $$\begin{array}{l} MWTP=-\frac{\partial L/\partial a}{\partial L/\partial y\left(h\right)\;} \end{array}$$

Of which:


(7) $$\begin{array}{l} \frac{\partial L}{\partial a}=-U_{h}g_{e}e_{a}+U_{e}e_{a}-\lambda y_{h}g_{e}e_{a}=U_{e}e_{a}-g_{e}e_{a}\left(U_{h}+\lambda y_{h}\right) \end{array}$$

Equation 8 is obtained by differentiating air pollution, health conditions, and working hours lost in Equation 7 and simplifying it as follows:


(8) $$\begin{array}{l} \frac{\partial L}{\partial a}=U_{e}\left[\frac{de}{da}-e_{m}\frac{\partial m}{\partial a}\right]\\ \;+\left[\frac{dh}{da}-\frac{\partial m}{\partial a}+g_{e}e_{m}\frac{\partial m}{\partial a}\right]\left[U_{h}+\lambda y_{h}\right] \end{array}$$

Combining Equations 5 and 8 produces Equation 9:


(9) $$\begin{array}{l} \frac{\partial L}{\partial a}=\frac{\partial m}{\partial a}\left(-\lambda p+U_{h}\right)+\frac{\partial h}{\partial a}\left(U_{h}+\lambda y_{h}\right)+U_{e}\frac{de}{da} \end{array}$$

The denominator of the MWTP can also be directly expressed as $\frac{\partial L}{\partial a}=\lambda$, and dividing by Equation 9 yields


(10) $$\begin{array}{l} MWTP=\left(p-\frac{U_{h}}{\lambda }\right)\frac{\partial m}{\partial a}-\frac{\partial h}{\partial a}\left(U_{h}+\lambda y_{h}\right)-\frac{de}{da}\frac{U_{e}}{\lambda } \end{array}$$

Expanding and simplifying the above equation yields the final Equation 11:


(11) $$\begin{array}{l} MWTP=\frac{\partial m}{\partial a}\left[p-\frac{U_{h}}{\lambda }\right]+\left(\frac{U_{h}}{\lambda }+y_{h}\right)\frac{\partial h}{\partial a}-\frac{de}{da}\frac{U_{e}}{\lambda } \end{array}$$

The first term on the right side of the equal sign in Equation 11 can be regarded as the cost of paying for healthcare under air pollution, $\frac{\partial m}{\partial a}$. The second term can be considered as the loss of labor efficiency due to air pollution, which can be reflected in the loss of work capacity due to illness or the opportunity cost due to the delay in working hours. This paper does not discuss the loss of utility owing to illness in other areas as a result of data limitations.

Combined with the above theoretical analysis, the empirical part of this study measured the health benefits of air quality in two main ways: according to health expenditure savings owing to improved air quality. First, personal out-of-pocket savings help improve the welfare of those who lack defenses against air pollution, improve consumption structure, reduce subsistence consumption, and release people from other forms of consumption. In addition, savings due to health insurance reimbursement bolsters the sustainability of the health insurance fund and protects the corresponding financial investment. Second, air quality improvement safeguards against the loss of working hours and the consequent loss of wages, raising the population's income in terms of opportunity costs.

## Data and Variables

### The Air Quality Improvement Process

The sample city in our study is a provincial capital city in Western China. In 2017, the GDP of the sample city was over 1,300 billion CNY, ranking in the top 10 of all cities in China, and the resident population was over 16 million by the end of 2017. The basic topography of the sample city is plain, and the sample city also has rich medical resources; in particular, the number of physicians and beds in medical institutions is ranked in the top five of all cities in China. We believe that this sample city could be a representative case in research on the relationship between air quality and health outcomes.

To measure the air quality background of the sample city, we collected a series of pollution indicators to illustrate the air quality improvement process, using descriptive statistics. The pollution indicators for the sample city in 2015–2017 are described in Table 1, where both NO_2_ and particulate pollution (PM_10_ and PM_2.5_) are above China's secondary annual average standard.^3^ Particulate pollution significantly exceeded the standard. Both PM_10_ and PM_2.5_ exceeded the secondary annual average standard by more than 20 μg/m^3^, about 30% of the days between 2015 and 2017 had mild pollution and above, about 10% had moderate pollution and above, PM_10_ exceeded the secondary 24-h average standard on 16% of the days, and PM_2.5_ exceeded the same 25% of the time. According to the sample city's 2016 Environmental Quality Bulletin, air quality was ranked in the lower to middle range of 74 cities nationwide.

**Table 1:** Sample city's air pollution status.

**Variable**	**(1)**	**(2)**	**(3)**	**(4)**	**(5)**	**(6)**
	**Mean**	**Std**.	**Min**.	**Max**.	**Unit**	**National annual standard**
**Panel A: Pollution indicators**						
AQI (Air Quality Index)	85.2923	46.6955	18.6534	369.8114	Dimensionless	Below 100 is good quality
SO_2_	14.0845	5.5222	4.3813	38.4656	μg/m^3^	60
NO_2_	48.9433	15.2567	14.9057	112.1062	μg/m^3^	40
CO	1.0021	0.3511	0.4112	2.5722	mg/m^3^	4
PM_10_	96.1556	60.3444	14.8712	451.4689	μg/m^3^	70
PM_2.5_	57.9911	39.1775	6.6555	290.8890	μg/m^3^	35
O_3_ 8 h maximum	127.7735	55.2521	22.0012	300	μg/m^3^	160
**Panel B: Air quality divided by AQI**						
Excellent air quality	0.2045	0.4056	0	1		AQI: 0–50
Good air quality	0.5367	0.5067	0	1		AQI: 51–100
Mild pollution	0.1863	0.3812	0	1		AQI: 101–150
Moderate pollution	0.0623	0.2446	0	1		AQI: 151–200
Heavy pollution	0.0366	0.1866	0	1		AQI: 200–300
Serious pollution	0.0039	0.0578	0	1		AQI: 300
**Panel C: Frequency of air pollutant over daily standard**						
SO_2_	0.0013	0.0011	0	1		SO_2_ > 150
NO_2_	0.0432	0.1856	0	1		NO_2_ > 80
CO	0.0002	0.0012	0	1		CO > 4
O_3_ 8 h maximum	0.2843	0.4556	0	1		O_3_ 8 h > 160
PM_10_	0.1655	0.3712	0	1		PM_10_ > 150
PM_2.5_	0.2521	0.4332	0	1		PM_2.5_ > 75

*Information on air pollution variables was obtained from the environmental data processing support provided by the Qingyue Open Environmental Data Center (https://data.epmap.org). The above table spans the period from 1st January 2015 to 31st December 2017, and the pollution standards are HJ 633-2012 and GB3095-2012*.

**Figure 1:**
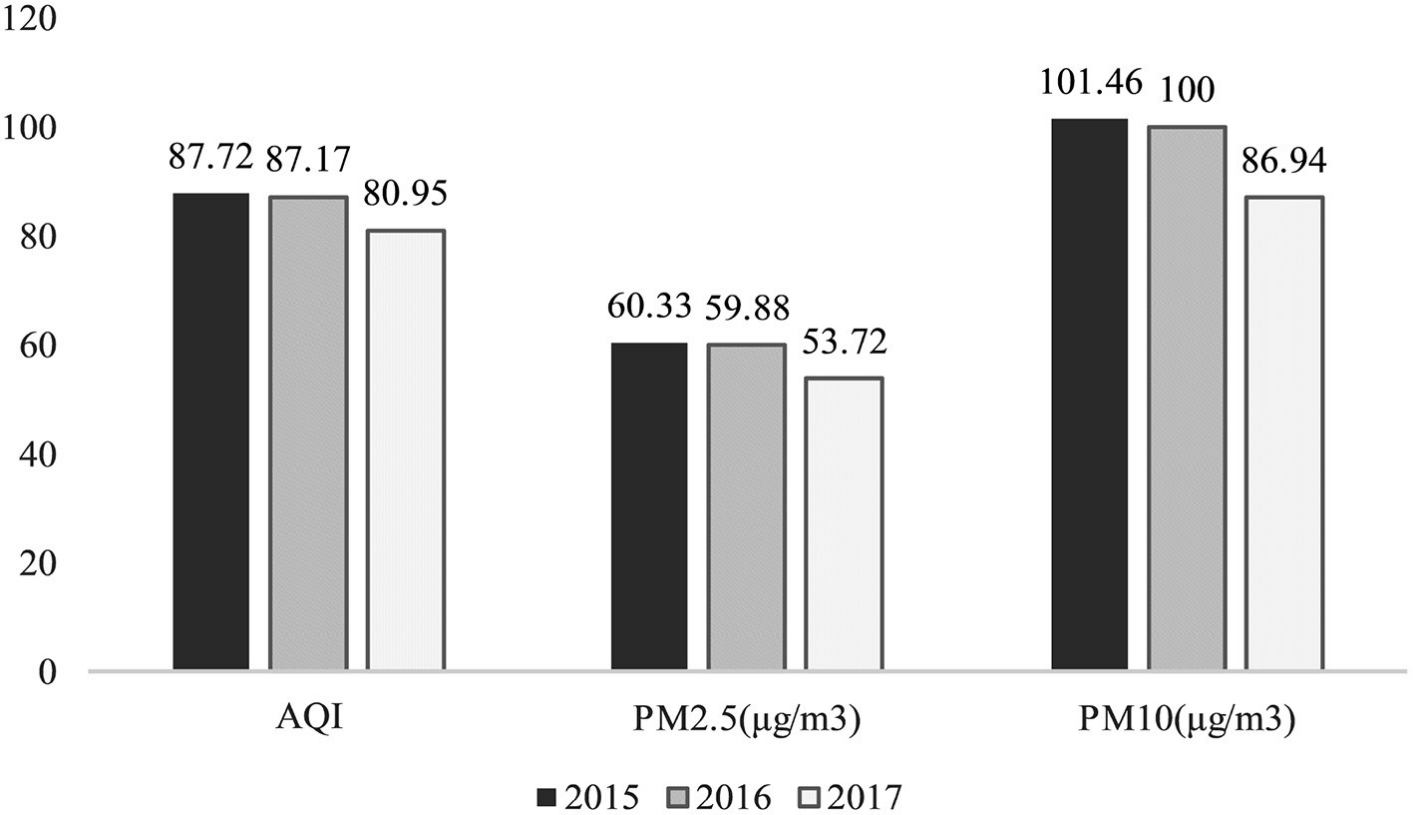
Annual air quality in sample city, 2015–2017.

According to national standards, the air quality of the sample city was not excellent. However, air quality dynamics are of greater interest given the city's topography is not conducive to pollution dispersion. The annual average of the major pollutant indicators for the sample city are shown in Figure 1, with the average AQI and average particulate pollution maintaining a downward trend over the 3 years. The decline observed in 2017 was greater than that in 2016, supporting this study's air quality improvement research.

### Measuring Patients' Exposure to Air Pollution

Although many studies have shown that various air pollutants, such as CO, NO_2_, SO_2_, could affect people's health (32, 37, 38), particulate pollutants are the major pollutants in the sample city. Notably, PM_2.5_ is incredibly harmful to humans (28). PM_2.5_ exceeded the secondary annual average standard by 38%; PM_2.5_ is also the leading cause of haze, which people can observe and perceive directly. In most studies, PM_2.5_ is used as a proxy measurement for air pollution (9, 18, 25), as in our research. Thus, we set PM_2.5_ as our key independent variable and control the other pollutants (CO/NO_2_/SO_2_/PM_2.5−10_) in our regression to enhance the efficiency of the regress model.

This study matched all air pollutants according to the patient's visit dates (from January 1 2016 to December 31 2017). The following two steps were performed: the daily air pollution concentrations index was first calculated on a forward-moving average and then matched to the patient's visit date to reflect the durational course of the air pollution effect. In keeping with the existing literature, a 7-day moving average window was used in this study, and the results were not sensitive to the moving average window (see Supplementary Material Appendix A). Second, to ensure the consistency of air quality and patient distribution, we obtained the concentrations of pollutants by averaging data from nine different monitoring stations located in the urban areas of sample city. In particular, we matched the patients in 78 public hospitals with air pollution day by day.

### Measuring Health Benefits

To measure health benefits, we used detailed health insurance reimbursement data from 78 public hospitals in a sample city. The database provides 563,383,685 expense details, including when each expense was incurred, cost, type of patient, type of patient reimbursement insurance, and diagnosed disease. The data included 869,781 patients with 1,766,690 visits. Health insurance claims were recorded for inpatient and special outpatient visits.^4^ However, our data did not include patients without health insurance or those who did not use their health insurance for payment (e.g., ordinary outpatients).

Referring to the existing literature (29–32), only samples from patients first diagnosed with a respiratory or circulatory disease, the diseases with the most direct effect, were used in the follow-up regression. The number of visits for circulatory and respiratory diseases was 213,804 and 313,840, respectively, accounting for 18 and 12% of the total number of visits, ranking first and third among the 22 major medical conditions coded as ICD-10.

Table 2 presents the descriptive statistics of the variables. Panel A is a variable description of the health insurance reimbursement data. Regarding total personal costs, patients' average total individual cost was CNY 10,616, and the per capita health insurance reimbursement expense was CNY 8,340, with reimbursement rates of more than 75%. Treatment fees accounted for a higher proportion than drug and consumable fees regarding the cost structure. Of the patients, 70.3% were inpatients, and 72.8% belonged to the Basic Medical Insurance for Urban Employee (UEBMI). In contrast, the other patients belonged to the Basic Medical Insurance for Urban and Rural Residents (URRBMI). Visit duration was obtained by subtracting the admission date from the discharge date, and the visit duration for the sample from special outpatient clinics was set to 1. The visit duration per capita was 8.55 days, and the average number of repeat visits per capita was 3.32, which means that all of the patients in our data, on average, will go to the hospital more than three times in 2 years. Panel B is a variable description from the air pollution and climate databases. For the pollution and climate indicators, we use a 7-day on-moving average.

**Table 2:** Descriptive statistics of variables.

**Variable**	**Observation**	**Mean**	**Std**	**Min**.	**Max**.
**Panel A: variables from health insurance reimbursement data**					
Total individual costs (Cost of visits)	527,645	10,616.4165	15,014.1904	690.0600	95,787.2031
Insurance reimbursement expenses	527,645	8,340.2325	10,174.8192	627.6041	62,383.4141
Individual out-of-pocket expenses	515,002	2,198.8969	5,476.5628	0.0001	38,426.0820
Cost of drug	527,645	3,867.8017	4,588.1423	184.0400	29,621.1660
Cost of consumables	378,692	2,055.3273	7,660.2320	1.9800	53,677.1289
Cost of treatment	414,051	6,575.6370	7,514.0128	18	48,493.2109
Patient type: inpatients	527,645	0.7034	0.4567	0	1
Insurance type: UEBMI	527,645	0.7275	0.4453	0	1
Visit duration (days)	527,645	8.5511	8.5832	1	46
Number of repeat visits	527,645	3.3163	2.9170	1	45
**Panel B: variables from air pollution database and climate database**					
7-day average surface air temperature (°C)	527,645	18.5581	7.8227	3.5000	32.9625
7-day average sunshine hours (h)	527,645	2.9586	2.0055	0.0000	9.7625
7-day average pressure (hPa)	527,645	951.2132	6.3978	939.1250	964.4250
7-day average relative humidity (%)	527,645	81.0924	5.6805	64.7500	93.2500
7-day average temperature (°C)	527,645	16.4359	7.0856	1.9375	29.0750
7-day average evaporation (mm)	527,645	1.7799	0.7850	0.6000	4.2875
7-day average PM_2.5_ (^μg/m^3^^)	527,645	58.4380	31.9735	14.1177	193.6603
7-day average wind speed (^m/s^)	527,645	1.3261	0.2084	0.7625	1.9125
7-day average thermal inversion intensity (°C)	527,645	0.8602	1.6251	0.0000	8.4879
7-day average sulphur dioxide (^μg/m^3^^)	527,645	14.0047	3.4600	7.3711	26.7350
7-day average NO_2_ (^μg/m^3^^)	527,645	49.8129	11.2201	24.2242	91.1488
7-day average CO (^mg/m^3^^)	527,645	1.0155	0.3064	0.6022	2.2103
7-day average O_3_ (^μg/m^3^^)	527,645	126.1352	43.0932	55.5000	238.2500
7-day average PM_2.5−10_ (^μg/m^3^^)	527,645	38.6782	19.0024	13.4486	114.5743

*Source: Health insurance reimbursement database of a provincial capital city. The authors calculated several indicators. Information on climate variables was obtained from publicly available data: National Greenhouse Data System data.sheshiyuanyi.com/WeatherData/*.

*For specific types of disease, the total per capita cost of respiratory diseases was CNY 10,601 and the per capita visit duration in days was 10.84. In contrast, it was CNY 11,136 and 7.52 days for circulatory diseases. Of the patients with respiratory diseases, 99.8% were inpatients, 55% had UEBMI, and the number of repeat visits was 2.5 in both years. Inpatients and special outpatients accounted for 50% of patients with circulatory diseases, respectively. In 84.8% of the patients with circulatory diseases, the insurance type was UEBMI and repeat visits were four in both years*.

## Empirical Methodology

Although air quality is a relatively exogenous variable for individual behavior, an individual's air pollution exposure can be endogenous. The presence of factors that affect both individual air pollution exposure and patient healthcare expenditure can lead to omitted variable bias. This study focuses on obtaining consistent estimates by controlling for fixed effects and using instrumental variables. For selecting the baseline model, we used the applied microeconometric method because we tried to solve the endogeneity problem to obtain causal inference results.^5^

### Controlling for Fixed Effects

The first method eliminates time-invariant omitted variables and time trends by including dummy variables as much as possible. In addition, hospital records use ICD-10 codes for disease types, which can be further subdivided into 6-digit codes for respiratory and circulatory diseases, reflecting differences on disease type and degree of illness.

Unlike other autonomous consumptions, inter-individual differences in public hospital medical costs mainly reflect disease type and hospital differences. The scope for individual patient decisions related to the expenses of visits is relatively small. Therefore, we controlled for hospital fixed effects and used ICD-10 6-digit codes corresponding to disease-type fixed effects to control for some patient differences to a certain extent. The year, month, and day of week fixed effects are also controlled to eliminate the effects of time trends and specific cyclical events (18).

### Instrumental Variables

Given that fixed effects cannot absorb the effects of factors such as possible short-term shocks, including workplace changes and unexpected public events that affect both patient air pollution exposure and visit costs (25), we add instrumental variables to the regressions. In this study, thermal inversion intensity was selected as the instrumental variable in the data context.

Thermal inversion is an atmospheric phenomenon in which the atmospheric temperature appears to be hotter at the top and colder at the bottom. The formation of an inversion layer hinders the diffusion of pollutants. This phenomenon does not directly affect humans, except through channels that affect pollution diffusion; therefore, inversion has become a widely used instrumental variable in air pollution studies (6, 16, 26, 33–36). In this study, thermal inversion data were obtained and processed in the NASA-MERRA2 database by referring to Sager (35) (see Appendix B for details).

Finally, the baseline regression equation used in this study is shown in Equation 12, where footer *I* denotes individuals and footer *t* denotes time, where *cost*_*it*_ includes the cost of the visit and the visit duration in days, both treated logarithmically. PM_2.5it_ is The average PM_2.5_ during the window period for each patient, *weather*_*it*_are climatic variables including days of sunshine, surface air temperature, air pressure, relative humidity, air temperature, evapotranspiration, and wind speed that can contribute to respiratory or circulatory diseases (28, 37, 38). *Pollution*_*it*_ represents the additional air pollution indicators shown in Table 2, including sulfur dioxide (SO_2_), nitrogen dioxide (NO_2_), carbon monoxide (CO), ozone (O_3_), and PM_2.5−10_ concentrations, with all of the above indicators taken as moving averages for the 7 days before the visit. *X*_*i*_ represents the remaining control variables, including patient type, insurance type, visit duration, and the number of repeat visits^6^; *hospital*_*i*_represents hospital fixed effects; *disease*_*i*_ represents the 6-digit ICD-10 coded disease fixed effects; and *T* represents the time fixed effects, such as weekends, months, and seasons. The first-stage regression equation is shown in Equation 13, where *thermal_iv*_*it*_ is the thermal inversion intensity, and ε_*it*_, μ_*it*_ is the residual term. All standard errors of the regressions are robust standard errors.


(12) $$\begin{array}{r} ln\left(cost_{it}\right)=\beta_{0}+\beta_{1}PM_{2.5it}+\lambda_{1}weather_{it}+\lambda_{2}pollution_{it}\\ +\phi X_{it}+hospital_{i}+disease_{i}+T+\varepsilon_{it} \end{array}$$

(13) $$\begin{array}{r} {pm2.5}_{t}=\alpha_{0}+\alpha_{1}{thermal\text{\_}iv}_{it}+\theta_{1}weather_{it}\\ +\theta_{2}pollution_{it}+\sigma X_{it}+hospital_{i}+disease_{i}+T+\mu_{it} \end{array}$$

## Empirical Results

### Baseline Results

Table 3 shows the baseline regression results. The first column presents the results of the first stage of the two-stage least squares (2SLS) regression, which shows a significant positive effect of thermal inversion intensity on PM_2.5_, as expected. The Kleibergen-Paap rk Wald F value of the first-stage regression is well above the critical value that satisfies the correlation requirement between the instrumental and endogenous variables, thus rejecting the weak instrumental variables hypothesis and indicating that the results of the 2SLS regression are reliable. The second and third columns show the effect of PM_2.5_ on patient visit costs and duration after applying the instrumental variables, with coefficients indicating that for every 10 μg/m^3^ decrease in PM_2.5_, visit costs for patients with respiratory and circulatory diseases would decrease by 16% and visit duration would decrease by 14%, both of which are significant at the 1% level. In concrete terms, for every 10 μg/m^3^ reduction in PM_2.5_, patients' average cost would be reduced by CNY 1,699 (USD 263.6), and the number of regular workdays lost would be reduced by 1.24 days.

**Table 3:** Impact of air pollution on the cost of visits and visit duration in days under 2SLS.

	**(1)**	**(2)**	**(3)**
	**First stage**	**Cost of visits**	**Visit duration**
7-day average PM_2.5_		0.0162^***^	0.0143^***^
		(0.0036)	(0.0034)
7-day average thermal inversion intensity	0.5375^***^		
	(0.0398)		
Patient type: inpatients	−0.1814^**^	1.1588^***^	2.3499^***^
	(0.0747)	(0.0718)	(0.0521)
Insurance type: UEBMI	0.2241^***^	0.0921^***^	0.1356^***^
	(0.0328)	(0.0259)	(0.0111)
Visit duration in days	0.0054^***^	0.0377^***^	
	(0.0015)	(0.0053)	
Number of repeat visits	0.0408^***^	−0.0057	0.0277^***^
	(0.0082)	(0.0040)	(0.0039)
Observation	528,051	527,645	526,789
Kleibergen-Paap rk Wald F-value	.	183.2	181.2
R square	0.9257	0.3647	0.5514
Climate variables	Y	Y	Y
Pollution variables	Y	Y	Y
Year fixed effects	Y	Y	Y
Monthly fixed effects	Y	Y	Y
Day of week fixed effect	Y	Y	Y
Disease fixed effects	Y	Y	Y
Hospital fixed effects	Y	Y	Y

*All standard errors of the regressions are robust*.

** *indicates significance at the 5% level and*

*** *indicates significance at the 1% level*.

*The cost of visits and visit duration in days were treated logarithmically. The unit for the 7-day average PM_2.5_ in the table is μg/m^3^. Visit type takes the value of 1 for inpatients, and insurance type takes the value of 1 for UEBMI. Among the control variables, the climate variables were sunshine days, average surface air temperature, average air pressure, average relative humidity, average air temperature, evaporation, and wind speed. Other pollution indicators included SO_2_, NO_2_, CO, PM_2.5−10_, and O_3_*.

Song et al. (15) are the only scholars to use an approximate database in China with medical insurance sampling data from Shanghai. They showed a 0.36% change in respiratory pulmonary-related medical costs for every 10 μg/m^3^ change in PM_10_ compared to the larger estimated coefficient in our study, which may be due to the more direct health hazards of PM_2.5_. Given that Song et al. (15) did not use instrumental variables to estimate, the results may underestimate the actual coefficients. Liao et al. (26) used micro-survey data without instrumental variables and found a 10 μg/m^3^ change in PM_2.5_, and a 2% change in health care costs; the regression results using instrumental variables are more consistent with our study, showing a 10 μg/m^3^ change in PM_2.5_, and a 17.1% change in health care costs. Our study's regression results are more consistent with Barwick et al. (25), who used China UnionPay credit card data and found that the effect of PM_2.5_ on hospital credit card spending with a 7-day lag was also a 16% change in costs for every 10 μg/m^3^ change in PM_2.5_.

### Robustness Check

Next, we perform a series of robustness checks to consider the effects of changes in the different factors on the benchmark regression results in Table 3.

#### Wind Speed Was an Alternative Instrumental Variable

Some studies have used wind speed as an instrumental variable for air pollution (25, 26, 34, 36). Thermal inversion central inhibited pollution dispersion by reducing atmospheric flow, wind speed also reflects the degree of atmospheric flow; the faster the wind speed, the faster the pollutant dispersion, and the lower the city's air pollution level. Our study's sample city is located on a plain, where topographical factors affect wind speed less. It is also suitable to choose wind speed as the instrumental variable of PM_2.5_. However, in our IV-alternative robustness check, only patients with the circulatory disease were tested, as respiratory disease, which has some association with wind speed, may avoid the exogenous hypothesis of the instrumental variable.

The regression results are presented in Table 4, which shows that wind speed significantly reduces PM_2.5_ concentration. The effects on the cost and visit duration are equally significant but with smaller coefficients than the results using thermal inversion intensity as an instrumental variable.

**Table 4:** Impact of air pollution to circulatory disease patients with alternative IV.

	**(1)**	**(2)**	**(3)**
	**First stage**	**Cost of visits**	**Visit duration**
7-day average PM_2.5_		0.0051^***^	0.0019^**^
		(0.0015)	(0.0009)
7-day average wind speed	−6.5303^***^		
	(0.1839)		
Patient type: inpatients	−0.1762^**^	1.2137^***^	2.3309^***^
	(0.0837)	(0.0664)	(0.0531)
Insurance type: UEBMI	0.1944^***^	0.0141	0.0942^***^
	(0.0547)	(0.0090)	(0.0111)
Visit duration in days	0.0056^**^	0.0318^***^	
	(0.0023)	(0.0047)	
Number of repeat visits	0.0674^***^	−0.0112^**^	0.0271^***^
	(0.0130)	(0.0045)	(0.0050)
Observation	314,087	313,840	313,287
Kleibergen-Paap rk Wald F-value		1265	1265
R square	0.9232	0.4301	0.7046
Climate variables	Y	Y	Y
Pollution variables	Y	Y	Y
Year fixed effects	Y	Y	Y
Monthly fixed effects	Y	Y	Y
Day of Week fixed effect	Y	Y	Y
Disease fixed effects	Y	Y	Y
Hospital fixed effects	Y	Y	Y

*All standard errors of the regressions are robust*.

** *indicates significance at the 5% level and*

*** *indicates significance at the 1% level*.

*The cost of visits and visit duration in days were treated logarithmically. The unit for the 7-day average PM_2.5_ in the table is μg/m^3^. Visit type takes the value of 1 for inpatients, and insurance type takes the value of 1 for UEBMI. Among the control variables, the climate variables were sunshine days, average surface air temperature, average air pressure, average relative humidity, average air temperature, and evaporation. Other pollution indicators included SO_2_, NO_2_, CO, PM_2.5−10_, and O_3_. Wind speed was no longer included as a climate-control variable*.

#### Difference-in-Difference Model

Because the data included patients with diseases other than respiratory and circulatory diseases, we used differences in disease type and air pollution between months to construct a difference-in-difference (DID) model. We matched patients pathologically affected by air pollution with patients unaffected to form the control group. Specifically, two diseases with a similar number of visits and cost per visit were selected using 3-digit ICD-10 codes, namely “bronchitis from respiratory diseases” and “arthritis from the musculoskeletal system and connective tissue diseases.^7^” Patients with bronchitis were assigned to the treatment group (treat = 1), and patients with arthritis were assigned to the control group (treat = 0).

The effect of PM_2.5_ on the cost of visits to patients with arthritis disease is first examined in Appendix C, Figure c1, and the results of the regression coefficients for different time windows are shown. As expected, no significant effect of PM_2.5_ was found for the various windows, ranging from 2 to 330 days, thus ensuring the integrity of the control group.

Next, a DID model was constructed to examine time-dependent changes in air pollution based on between-group differences in PM_2.5_. This study examines changes in the effects of air pollution on the two diseases mentioned above from August 2016 to March 2017, using July 2016 as the base time group.^8^ The regression equation is given by Equation 14.


(14) $$\begin{array}{l} \ln\left(fee_{it}\right)=\beta_{0}+\beta_{t}\overset{2017/3}{\underset{2016/7}{\sum }}Treat_{it}^{*}month_{t}+\lambda_{1}weather_{it}\\ +\lambda_{2}pollution_{it}+\phi X_{it}+hospital_{i}+disease_{i}+T+\varepsilon_{it} \end{array}$$

**Figure 2:**
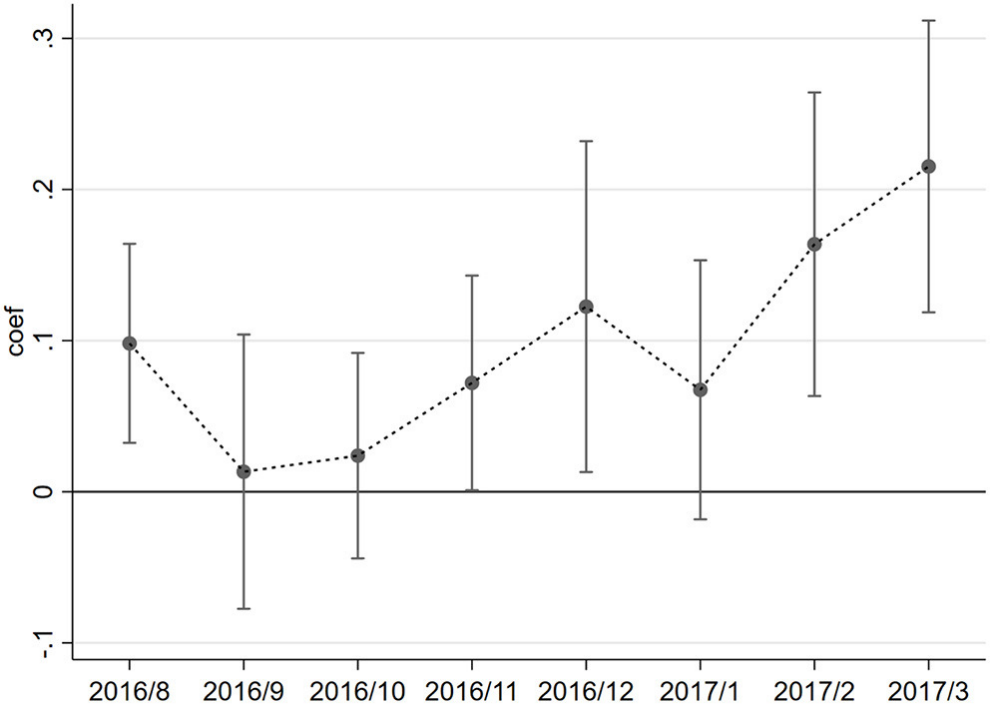
Time trend of the cost variation factor by disease type.

**Figure 3:**
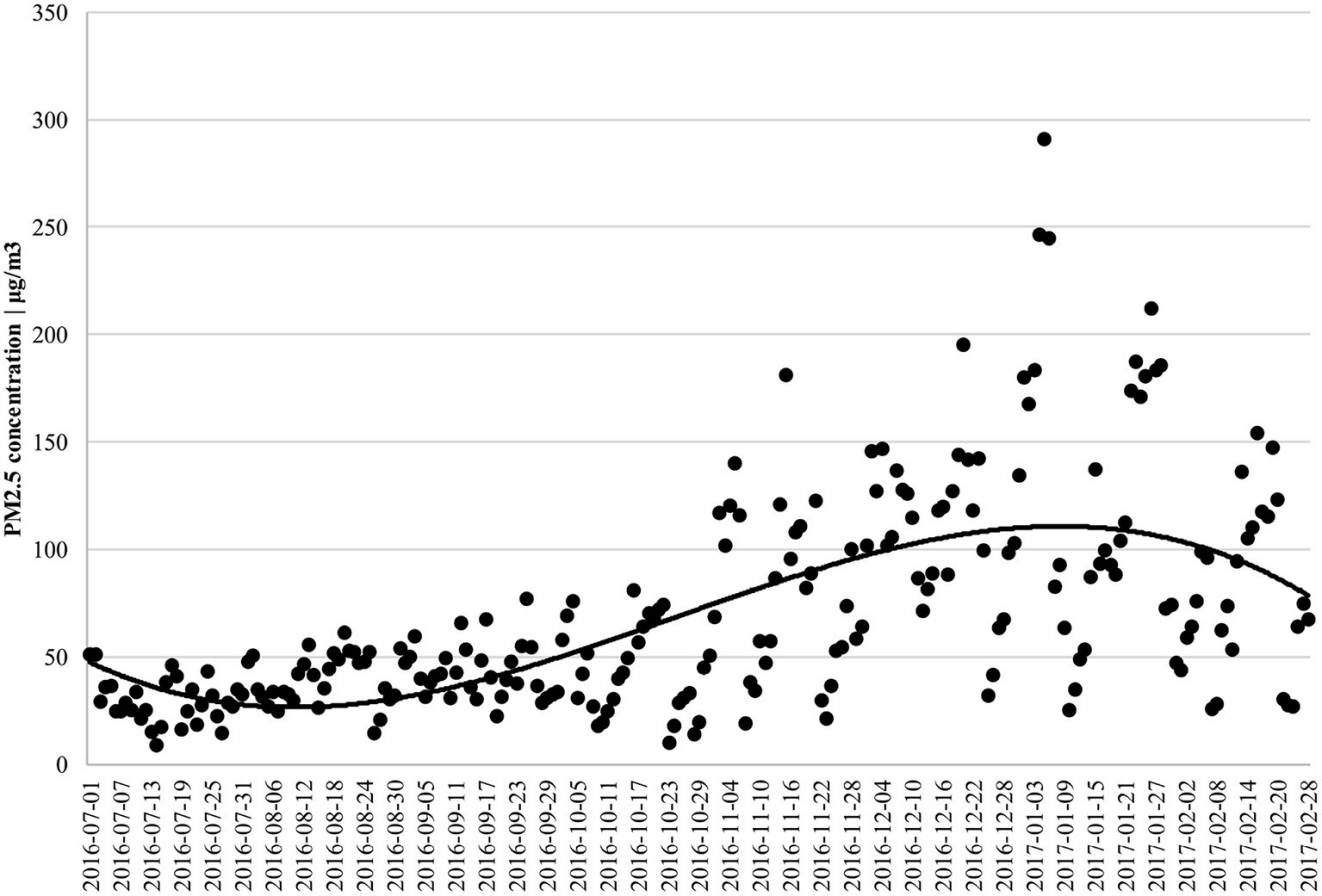
Time trend of PM_2.5_ in sample city.

The variation in the coefficient β_*t*_ within a month is shown in Figure 2. No significant difference was found in the overall effect of PM_2.5_ between disease types from August to October when air quality was better (Figure 3). From November to March of the following year, PM_2.5_ had a significantly greater effect on the cost of visits for patients with bronchitis than patients with arthritis since the bad air quality showed as Figure 3, supporting the positive finding drawn from the baseline regression results. This result also suggests seasonal differences in the impact of PM_2.5_ on patients with respiratory diseases, which may result in higher medical costs during seasons with high air pollution.

#### Other Factors That May Affect Identification

In addition to solving the problem of potential endogeneity, three issues may lead to biased identification. The first issue is the variation in air quality. The core explanatory variable PM_2.5_ differs only temporally between samples in this study. If two patients visited the hospital on similar days, the difference in their exposure to PM_2.5_, after the moving average, would be further reduced. Therefore, we only retained patients who visited Tuesdays, Thursdays, and Saturdays to amplify the differences in the core explanatory variables. The regression results are shown in columns (1) and (2) of Table 5, with robust regression coefficients and significance compared with Table 3.

**Table 5:** Robustness check.

	**(1)**	**(2)**	**(3)**	**(4)**	**(5)**	**(6)**
**Variables**	**Limited in next-day sample**		**Limited in local patients**		**Add warning variable**	
	**Cost of visit**	**Visit duration**	**Cost of visit**	**Visit duration**	**Cost of visit**	**Visit duration**
7-day average PM_2.5_	0.0163^***^	0.0163^***^	0.0093^***^	0.0138^***^	0.0047^***^	0.0015
	(0.0050)	(0.0053)	(0.0025)	(0.0024)	(0.0015)	(0.0009)
Number of 7-day AQI exceedances					0.2658	0.2095^*^
					(0.1720)	(0.1246)
Patient type: inpatients	1.1677^***^	2.3470^***^	0.8360^***^	2.5209^***^	1.2111^***^	2.3291^***^
	(0.0759)	(0.0568)	(0.0107)	(0.0053)	(0.0661)	(0.0531)
Insurance type: UEBMI	0.0855^***^	0.1357^***^	0.1327^***^	0.1507^***^	0.0137	0.0939^***^
	(0.0271)	(0.0114)	(0.0050)	(0.0045)	(0.0089)	(0.0110)
Visit duration in days	0.0383^***^		0.0425^***^		0.0318^***^	
	(0.0056)		(0.0007)		(0.0047)	
Number of repeat visits	−0.0054	0.0280^***^	0.0007	0.0273^***^	−0.0113^**^	0.0269^***^
	(0.0044)	(0.0040)	(0.0006)	(0.0005)	(0.0046)	(0.0050)
Observation	218,021	217,635	135,236	134,921	313,840	313,287
R square	0.3728	0.5513	0.4093	0.5972	0.4275	0.7031
Control variables	Y	Y	Y	Y	Y	Y
Time fixed effect	Y	Y	Y	Y	Y	Y
Disease and hospital fixed effects	Y	Y	Y	Y	Y	Y
Kleibergen-Paap rk Wald F-value	81.30	79.62	759.3	764.7	167.3	169.0

*All the standard errors of regressions are robust standard errors*.

* *indicates significance at the 10% level*,

** *indicates significance at the 5% level, and*

*** *indicates significance at the 1% level*.

*Year, month, and week fixed effects are controlled for as time fixed effects, as in Table 3. Cost of visit and visit duration are treated logarithmically. Control variables include days of sunshine, average surface air temperature, average air pressure, average relative humidity, average air temperature, evaporation, and wind speed. Other pollution indicators include SO_2_, NO_2_, CO, PM_2.5−10_, and O_3_. Wind speed is no longer included among the climate control variables in columns (5) and (6)*.

Second, regarding the problem of people migration, if patients travel to the sample city from other cities to treat their illness, their exposure to air pollution may not coincide with the sample city in terms of time and space, and directly matching the pollution and climate levels in the sample city may bias the estimation results. Fortunately, patients in the database are those with local health insurance, and their workplaces and residences should be located in the sample city for an extended period; however, as the sample city is a provincial capital city, several of the larger hospitals may have admitted more patients from outside the core urban area. Because of this, we excluded the top ten hospitals in terms of visits; the remaining hospitals have a large local population in terms of proximity. The regression results are shown in columns (3) and (4) of Table 5 and are equally robust.

The third issue is the effect of avoidance behavior. Some residents may reduce their outdoor activities to minimize exposure to negative effects (3, 39). As outdoor PM_2.5_ may not reflect the proper air pollution levels to which patients are exposed, this paper refers to Janke (3) to add an early warning dummy variable for air pollution as the control variable for the effect of avoidance behavior. Daily, people may pay more attention to AQI values than PM_2.5_, as AQI is more straightforward for day-to-day comparisons. AQI is also better at reflecting the effect of early warnings; therefore, we calculated the number of times AQI reached pollution levels 7 days before the patient's visit. To determine the endogeneity of AQI similar to PM_2.5_, we used wind speed as the instrumental variable of AQI. Only patients with circulatory diseases were retained owing to relationship between respiratory diseases and wind speed. Overall, thermal inversion intensity and wind speed were used as instrumental variables for PM_2.5_ and the number of and AQI exceedances, respectively. Columns (5) and (6) of Table 5 present the results. A decrease in the PM_2.5_ coefficient is observable, including the AQI exceedance count, but the coefficient is still significant for the cost of visits.

### Heterogeneity Analysis

Different types of patients and those with other characteristics may exhibit different responses to air pollution. Patients themselves may self-categorize them based on self-identification differences (7), which can be observed through differences in the impact of air pollution on visit costs.

Some studies grouped samples according to commonly used demographic information, such as sex, age, and income (40–42). Patient disease type heterogeneity has also been explored because of limited demographic information for micro-individuals (3, 7, 43). The inability of the data to differentiate patient characteristics through more detailed demographic variables limited this study. However, relying on the wealth of information about disease types provided by the ICD-10 codes, we analyzed heterogeneity in terms of disease, patient, and cost types.

#### Disease Types

Differences in the effect of PM_2.5_ were examined by differentiating disease types into acute and chronic diseases based on ICD-10 codes.^9^ It is easier to distinguish respiratory system diseases according to the disease name.^10^ Columns (1)–(3) of Table 6 show the regression results, with a significant effect of PM_2.5_ on the cost of visits for patients with respiratory diseases under all disease types. These values were slightly higher than the baseline regression in Table 3. PM_2.5_ has a greater effect on patients with chronic diseases, probably because patients with chronic diseases experience long-term sub-health conditions and are more sensitive to air pollution, including short-term exposure, which is more likely to lead to disease recurrence.

**Table 6:** Impact of PM_2.5_ on cost of visit: heterogeneity analysis.

**Variables**	**(1)**	**(2)**	**(3)**	**(4)**	**(5)**	**(6)**	**(7)**	**(8)**	**(9)**
	**Respiratory diseases all patients**	**Acute**	**Chronic**	**Circulatory disease all patients**	**Special outpatients**	**Inpatients**	**Drugs**	**Consumables**	**Treatment**
7-day average PM_2.5_	0.0185^***^	0.0081^***^	0.0142^***^	0.0123^**^	0.0203	0.0137^**^	0.0155^***^	0.0121^*^	0.0201^***^
	(0.0027)	(0.0030)	(0.0024)	(0.0051)	(0.0439)	(0.0067)	(0.0040)	(0.0063)	(0.0038)
Observation	213,804	46,370	98,684	313,840	156,058	157,742	527,645	379,039	414,451
R square	0.2647	0.3168	0.3330	0.4158	−0.0957	−0.0239	0.1668	0.2000	0.4757
Respiratory disease	Y	Y	Y				Y	Y	Y
Circulatory disease				Y	Y	Y	Y	Y	Y
Control variables	Y	Y	Y	Y	Y	Y	Y	Y	Y
Time fixed effect	Y	Y	Y	Y	Y	Y	Y	Y	Y
Disease & Hospital fixed effects	Y	Y	Y	Y	Y	Y	Y	Y	Y
Kleibergen-Paap rk Wald F-value	785.5	265.5	453.6	71.77	1.150	874.8	183.2	1,370	683.6

*All standard errors of the regressions are robust*.

* *indicates significance at the 10% level*,

** *indicates significance at the 5% level, and*

*** *indicates significance at the 1% level*.

*Year, month, and week fixed effects are controlled for as time fixed effects, as shown in Table 3. The cost of the visit was logarithmically treated. The control variables included patient type, insurance type, visit duration, number of repeat visits, days of sunshine, average surface air temperature, average air pressure, average relative humidity, average air temperature, evaporation, and wind speed. Other pollution indicators included SO_2_, NO_2_, CO, PM_2.5−10_, and O_3_*.

#### Patient Type

Since most patients with respiratory diseases in the data were inpatient visits and precisely 50% of patients with circulatory diseases were special outpatients and 50% inpatients, the heterogeneity analysis distinguished visit types mainly for patients with circulatory diseases. Columns (4)–(6) of Table 6 present the regression results, column (4) shows that PM_2.5_ has a significant effect on the overall cost of circulatory disease visits, with a slightly lower coefficient than a respiratory disease. Further differentiation of patient types shows that PM_2.5_ has a statistically more significant effect on inpatients. The non-significant impact on special outpatients is mainly because special outpatients with circulatory diseases are specifically prescribed regular hospital visits; therefore, the cost of visits is less affected by short-term environmental changes.

#### Cost Type

According to the treatment method, the individual costs can be divided into drugs, consumables, and treatment costs. Columns (7)–(9) of Table 6 show the regression results, indicating that PM_2.5_ has a more significant effect on drug and treatment costs, indicating that air pollution mainly increases the cost of drug and treatment and has a relatively small impact on treatment modalities such as surgery. In the current Chinese healthcare reform, improving air quality will also help reduce the proportion of drugs.

## Further Analysis

Further analysis discusses three main aspects: first, to examine the differences in the impact of PM_2.5_ on health care expenditures via different payment methods and with varying types of insurance, that is, to explore the moderating role of health insurance in the impact of PM_2.5_ on the cost of visits, which entails an empirical analysis of the interaction between health insurance reimbursement rates and air pollution in the theoretical model; second, to examine the non-linearity of PM2.5 with the threshold identification method; and last, to measure the health benefits of air quality improvement, based explicitly on regression coefficients and sample data.

### The Impact of Health Insurance Reimbursement

Based on detailed information about patients' payment types, we can distinguish the costs associated with reimbursement and out-of-pocket expenses, where the former are paid by the health insurance coordination fund and the latter are the portion of the total visit cost personally borne by the patient after removing the part of the health insurance pays. Hence, the out-of-pocket cost can reflect patients' personal payment pressure due to the cost of the visit. Panels A, columns (2) and (3) in Table 7 show the effects on reimbursement and out-of-pocket costs, respectively. The regression coefficients are all significant at the 1% level. Converted to specific amounts, for every 10 μg/m^3^ decrease in PM_2.5_, the per-patient savings on average health insurance expenditure will be CNY 1,501 (USD 233), and the actual personal health care burden will be reduced by CNY 368 (USD 57).

**Table 7:** Impact of PM_2.5_ on cost of visit: further analysis, consider reimbursement.

	**Total cost**	**Reimbursement of expenses**	**Out-of-pocket expenses**
**Panel A**	**(1)**	**(2)**	**(3)**
7-day average PM_2.5_	0.0162^***^	0.0176^***^	0.0277^***^
	(0.0036)	(0.0039)	(0.0095)
Observation	527,645	528,046	514,684
R square	0.3647	0.3458	0.2184
Kleibergen-Paap rk Wald F-value	183.2	321.4	186.4
**Panel B**	**(1)**	**(2)**	**(3)**
7-day average ${\text{PM}}_{2.5}^{*}$ UEBMI	0.0138^***^	0.0131^***^	0.0202^***^
	(0.0014)	(0.0013)	(0.0038)
7-day average PM_2.5_	−0.0055	−0.0031	−0.0041
	(0.0034)	(0.0038)	(0.0085)
UEBMI	−0.7076^***^	−0.5950^***^	−1.5193^***^
	(0.0688)	(0.0678)	(0.2225)
Observation	527,645	528,046	514,684
R square	0.3444	0.3344	0.2144
Kleibergen-Paap rk Wald F-value	151.1	151.1	155.3
Control variables	Y	Y	Y
Time fixed effect	Y	Y	Y
Disease and hospital fixed effects	Y	Y	Y

*All standard errors of the regressions are robust*.

*** *indicates significance at the 1% level*.

*Year, month, and week fixed effects are controlled for as time fixed effects, as shown in Table 3. The cost of the visit was logarithmically treated. The control variables included days of sunshine, average surface air temperature, average air pressure, average relative humidity, average air temperature, evaporation, and wind speed. Other pollution indicators included SO_2_, NO_2_, CO, PM_2.5−10_, and O_3_*.

The following section examines how PM_2.5_ impacts visit costs based on insurance reimbursement. Such differences may arise from moral hazard issues related to these two aspects of health insurance. First, patients expect lower treatment costs after purchasing health insurance–known as “ex-ante moral hazard”–and thus needlessly consume public resources. Second, medical personnel may use different treatments according to different reimbursement types, ultimately reflected in differences in medical costs (46).

This study's data set showed two patient classifications by insurance type: basic medical insurance for urban employees (UEBMI) and basic medical insurance for urban and rural residents (URRBMI). The reimbursement rate of UEBMI is higher than that of URRBMI, providing an opportunity to identify the second moral hazard problem in this study. The regression results of the interaction term between PM_2.5_ and reimbursement type are shown in Panel B of Table 7. The regression coefficients of the interaction term under different payment methods are significantly positive, indicating that PM_2.5_ has a significantly higher positive effect on health care expenditures for UEBMI patients than URRBMI patients. This result may be due to several factors. On the one hand, most urban workers with stable jobs and higher incomes are more willing to consume higher medical expenditures; however, it may be that the reimbursement rate for urban workers' health insurance is higher, and physicians differentiate treatment and medication based on insurance type, making the visit more expensive. We believe that the latter is more critical because patient characteristics have limited decision-making power concerning the cost of the visit, as the attending physician largely determines the treatment mode and cost. This result, therefore, provides potential evidence for the existence of the “induced demand” phenomenon.

### Non-linearity Identification of PM_2.5_

Although we identified that air pollution significantly increased health costs, policymakers need to consider not only the greatest degree to reduce the medical cost but also economic development opportunities; if the impact of air pollution on the cost of medical care is non-linear, it is more conducive for policymakers to find an appropriate point to balance the relationship between the two.

Ostro (47) was the first to focus on a threshold for the effect of winter air pollution on mortality in London, and the discussion of non-linearity has been addressed in many follow-up studies. Most of the identification methods in the literature fall into two categories: first, transforming continuous air pollution variables into discrete ones (18, 41) to observe differences in the effects of various levels of air pollution, and second, conducting an air pollution value with a threshold dummy variable and identifying the impact of interaction regression (7, 36).

**Figure 4:**
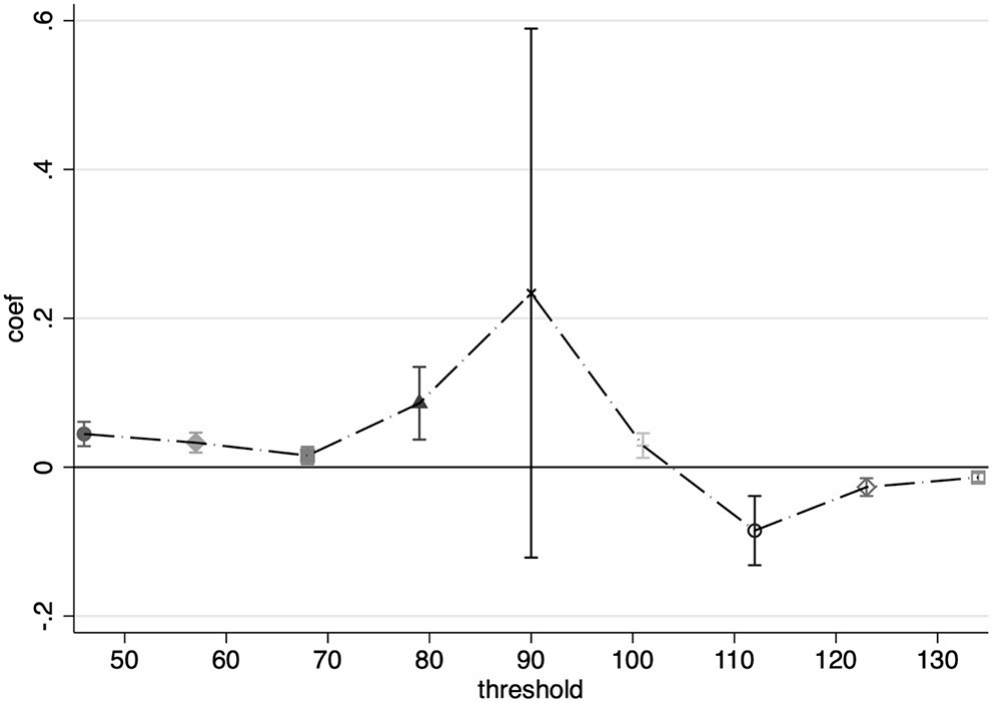
Non-linearity of PM_2.5_.

In this study, the second method of identification was more general. First, a dummy variable is generated to determine whether the PM_2.5_ concentration exceeds the potential threshold. A series of dummy variables are constructed based on the distribution of PM_2.5_ in the data, starting from 50 to 130 μg/m^3^ in steps of 10 μg/m^3^; if the current PM_2.5_ exceeds the threshold, the dummy variable is 1; otherwise, it is 0. The regression equation is shown in Equation 15, where β_*j*_ is the coefficient of concern, and the coefficient represents the difference in the effect of air pollution above the threshold compared to the effect below the threshold. Figure 4 shows the regression results at the different thresholds.


(15) $$\begin{array}{r} \ln\left(fee_{it}\right)=\beta_{0}+\beta_{j}PM_{2.5it}^{*}Threshold_{j}\\ +{\beta_{2}PM}_{2.5it}+\beta_{3}Threshold_{j}+\lambda_{1}weather_{it}+\lambda_{2}pollution_{it}\\ +\phi X_{it}+hospital_{i}+disease_{i}+T+\varepsilon_{it} \end{array}$$

Figure 4 reflects a marginal increasing effect of air pollution in concentrations of PM_2.5_, below 90 μg/m^3^, in the interval of 50–80 μg/m^3^ where the pollution index is concentrated. After reaching a 90 μg/m^3^, the non-linear effect of the marginal increment tends to disappear.

### Back-of-the-Envelope: Measuring the Health Benefits of Air Quality Improvement

The health benefits of air quality improvement include mortality, morbidity, medical visit costs, defensive consumption costs, work time, and productivity (40). In the existing literature, numerous studies examine mortality and morbidity; they also provide economically meaningful measures, such as the reduction of pollution to a certain level, the number of visits (3), the magnitude of the decline in deaths (40), or defensive consumption reduction (18, 25). This study focuses on measuring the health benefits of air quality improvement in terms of healthcare cost savings on the cost of visits and the reduced loss of labor income corresponding to visit duration.

The economized health benefits measured in this study based on the available data are a conservative estimate. Three aspects reflect the conservativeness of the estimation. First, the study sample only includes information about patients who have received health insurance reimbursement, and the data can only observe the population who have incurred health care behavior. The data cannot observe the impact of air pollution on the population that does not seek medical treatment. Second, there is a starting line for public health insurance reimbursement. The data mainly includes relatively high-cost patients, excluding general outpatients with relatively minor medical expenses; thus, the measured economic benefits may lower the real benefits. Third, the disease types of interest in this study are limited to respiratory and circulatory diseases. However, air pollution may also have pathological effects on other diseases such as mental illnesses (41). However, to ensure the reliability of the regression coefficients, only the two predominant diseases were included in the measurement.

The health benefits are calculated as follows:

Health gain for one unit of PM_2.5_ reduction

= (medical cost savings for one unit of PM_2.5_ reduction + wage loss reduction for one unit of PM_2.5_ reduction) × (1 + morbidity factor)

Table 8 shows the calculation process and results, with a conservative health gain of over CNY 546 million (USD 84.9 million) per year for each 10 μg/m^3^ reduction in PM_2.5_. Based on the existing national secondary annual average PM_2.5_ of 35 μg/m^3^, a reduction in the current annual average PM_2.5_, which would result in an annual health gain of at least approximately CNY 1.278 billion (USD 198 million) or about 18% of the city's 2016 environmental investment.^11^ Using the impact factor for reimbursement costs and per capita, health insurance reimbursement costs would result in at least CNY 1.032 billion (USD 160 million) in health insurance savings in 1 year or about 10.34% of the city's 2016 health insurance pooled fund expenditure.^12^

**Table 8:** Health benefits of measured air quality improvements.

**Types**	**Coef. of visit cost**		**Per capita visit cost**	**Annual visits**	**Morbidity factor**	**Medical cost savings per 10 μg/m^**3**^ reduction in PM_**2.5**_**	**Total**
Cost of respiratory disease visits	0.018		116,01	121,300	0.0015	253,678,711.31	546,300,926.75
Cost of circulatory disease visits	0.012		111,36	196,238	0.0004	262,341,658.87	
	**Coef. of visit duration**	**Average number of visit days**	**Per capita daily wage**	**Working population**	**Morbidity factor**	**Reduction in lost wages per 10** **μg/m**^**3**^ **reduction in PM**_**2.5**_	
Respiratory disease patients' wage loss	0.02	11	98.36	970,40	0.0015	21,017,559.06	
Circulatory disease patients' wage loss	0.008	7.5	98.36	156990.4	0.0004	9,262,997.51	

*The regression coefficients of visit cost and visit duration were obtained through regression, and the sample average PM_2.5_ concentration, sample disease per capita visit cost, and sample disease per capita for visit duration were obtained from the data description; the annual number of visits for the sample disease in 2016 was obtained using the sum of visit records; the sample city per capita daily wage was sourced from the 2016 statistical bulletin on the development of human resources and social security in the city: urban per capita disposable income was CNY 35,902, while the converted average daily income was CNY 98.36 per day; the average share of the working population was set at 80% according to the average share of the working-age population, and the coefficient of the effect of PM_2.5_ on the morbidity of the sample diseases was obtained from Qiu et al. (32) and Li et al. (49) to determine the first conservativeness problem*.

## Conclusions

Identifying the health effects of air pollution is a hot research topic in environmental and health economics, and measuring the health benefits of air quality improvement is of sufficient relevance in the context of the country's ongoing efforts to tighten air environment management. In this study, through a theoretical analysis of the health stock model, the impacts of air pollution on medical expenditures and visit duration were identified, and the health benefits to be gained from air quality improvement were estimated. The medical insurance reimbursement system data matched with daily pollution information provide a better identification setting in this study, which uses PM_2.5_ as the main proxy for air pollution, addresses endogeneity in identification as much as possible by controlling for fixed effects and employing thermal inversion intensity as an instrumental variable for air pollution and selects 7 days as a moving average window for PM_2.5_ based on sensitivity analysis. The baseline model results showed that for every 10 μg/m^3^ decrease in PM_2.5_, patients' health care expenditure would decrease by 16%. Visit duration would be reduced by 14%, corresponding to an average reduction in patients' health care costs of CNY 1,699 (USD 263.6) and a reduction of 1.24 days in normal working hours lost.

In this study, robustness checks were conducted in several ways, including replacing wind speed with an instrumental variable, constructing a DID model, changing the data structure, and adding AQI warning variables. Heterogeneity analysis revealed that PM_2.5_ has a more significant effect on visit costs for patients with chronic respiratory disease and circulatory inpatients and that PM_2.5_ more significantly affects the cost of drugs and treatment than the cost of consumables. Further analysis found that air quality improvement can reduce the burden on both health insurance funds and patients themselves, as demonstrated by the finding that for every 10 μg/m^3^ reduction in PM_2.5_, each patient will save 1,501 CNY (USD 233) on average health insurance expenditure. The actual personal burden of medical expenses will be reduced by 368 CNY (USD 57). Health insurance reimbursement has a moderating effect on the impact of air pollution on medical costs, with patients with higher reimbursement rates facing higher healthcare expenditures. This result may reflect induced demand from the supply side of healthcare. There also exists a non-linear relationship between PM_2.5_ and medical expenditure for PM_2.5_ concentration under 90 μg/m^3^. Finally, relying on regression coefficients and sample data, we found that for every 10 μg/m^3^ reduction in PM_2.5_, the city's health benefits for respiratory and circulatory patients would be CNY 546 million (USD 84.9 million) and a reduction in the PM_2.5_ concentration to the annual average secondary standard of 35 μg/m^3^ would result in annual health benefits of approximately CNY 1.278 billion (USD 198 million) or ~18% of the city's annual environmental investment, while savings on health care expenditures would be CNY 1.032 billion (USD 160 million) or about 10.34% of the city's annual health care pooled fund expenditure.

For the policy implications of the paper, first, we analyze the relationship between air quality and medical cost. However, our research is a case study based on a representative city; it could be a mode reference on assessing the public health benefit of environment quality improvement. Second, identifying the outcome of air pollution is a hot topic for interdisciplinary research in environmental and health economics. Measuring the health benefits of air quality improvements also provides a useful complement to existing research in the context of the country's ongoing air environment management. Third, we believe that air pollution has additional economic costs beyond health costs, particularly the induced demand and non-linearity shown in the impact process, which will expand the traditional scope of assessing environmental investment.

This study had several limitations. First, the data were from one city; the findings are limited to the sample city and cannot be generalized to the whole country. Second, our data cover only 2 years, from January 1, 2016, to December 31, 2017, representing a relatively short time frame and, consequently, cannot measure the long-term effects of air quality improvement. Third, our research is limited to the data of the medical insurance system and lacks multi-source data, which may affect the scientificity of the results. We will find more data sources to complement our research in future studies. We will continue to focus on policy estimates of air quality to create a more comprehensive air quality assessment.

## Data Availability Statement

The raw data supporting the conclusions of this article will be made available by the authors, without undue reservation.

## Author Contributions

HX: data curation, validation, original draft preparation, and software. DL: validation, data curation, writing, reviewing, and editing. SM: conceptualization and methodology. JZ: supervision, visualization, methodology, and editing. All authors contributed to the article and approved the submitted version.

## Funding

This work was supported by the 111 Project (grant number B16040), National Natural Science Foundation of China (grant numbers 71922015 and 71773075), and National Ten Thousand Talent Program Young Top-notch Talent Plan.

## Conflict of Interest

The authors declare that the research was conducted in the absence of any commercial or financial relationships that could be construed as a potential conflict of interest.

## Publisher's Note

All claims expressed in this article are solely those of the authors and do not necessarily represent those of their affiliated organizations, or those of the publisher, the editors and the reviewers. Any product that may be evaluated in this article, or claim that may be made by its manufacturer, is not guaranteed or endorsed by the publisher.

## Footnotes

^1^Available online at: http://www.jingbian.gov.cn/gk/zfwj/gwywj/41211.htm?tdsourcetag=s_pcqq_aiomsg (accessed January 18, 2021).

^2^Available online at: http://www.xinhuanet.com/politics/2018lh/2018-03/17/c_137045643.htm (accessed October 18, 2020).

^3^The national concentration limits for basic items of ambient air pollutants are divided into annual average, 24-h average and 1-h average. The different averaging times are divided into primary and secondary standards, and the values for primary standards are lower than those for secondary standards. Each indicator's exceedance frequency is compared to the secondary 24-h average standard.

^4^Special outpatients are patients with certain prescribed chronic diseases who are still eligible for reimbursement under the basic social health insurance when they visit public hospitals' outpatient departments, while ordinary outpatients are not eligible for reimbursement under the basic social health insurance. The special outpatient category includes 22 diseases, such as coronary heart disease, hypertension III, and diabetes mellitus.

^5^We also believe that the machine learning is more frontier method to analyze this question, especially for the non-linearity identification (48). In comparison, the machine learning method emphasize the micro perspective of the deep relationship between air pollution and illness process in sample individual perspective, we focused on the effect of air pollution in a comprehensive level, applied micro-econometric method would be better to capture the macro relationship and calculate the health benefit of air quality improvement.

^6^The patient type includes inpatient and special outpatient, the insurance type includes UEBMI and URRBMI. The visit duration in days is no longer included among the control variables when it is treated as dependent variable.

^7^The sample contains 15,659 visits for bronchitis, with a per capita visit cost of CNY 5,224, and 16,457 visits for arthritis, with a per capita visit cost of CNY 4,177.

^8^Period from August 2016 to March 2017 covered an enough variation of air quality and a consistent time section during the time window of our data. As Figure 3, in July 2016, a pretty good air quality month in 1 year, PM_2.5_ was almost under 50 μg/m^3^. Thus, we use July 2016 as the base time group.

^9^The effect examined is the different between disease, rather than the effect between short- and long-term of air pollution (44, 45). Given this study's regression design, a longer time period can be chosen to reflect the long-term effects of air pollution, but it will reduce the instrumental variables' exogeneity (see Appendix A); despite the data's high temporal frequency, it is still less than a 2 year time span, so this study mainly examines the effects of short-term air pollution exposure.

^10^Acute diseases include mainly the following codes: J00, J01, J02, J03, J04, J05, J06, J20, J21, J22, J45, J46, J47. Chronic diseases include J30, J31, J32, J33, J34, J35, J36, J37, J38, J39, J40, J41, J42, J44, J60, J61, J62, J63, J64, J65, J66, J67, J98.

^11^The city invested more than CNY 7 billion in environmental protection in 2016, according to data from the city's 2016 Environmental Quality Bulletin.

^12^The city's consolidated fund expenditure totaled CNY 9.984 billion in 2016, according to data from the city's 2016 Human Resources and Social Security Business Development Statistics Bulletin.
