# Supplementary material for: Health Benefits of Air Quality Improvement: Empirical Research Based on Medical Insurance Reimbursement Data
Source: Front Public Health. 2022 Mar 3;10:855457. doi: 10.3389/fpubh.2022.855457 (PMC8927649; doi:10.3389/fpubh.2022.855457)
Supplement: Supplementary file 1 [file Data_Sheet_1.pdf]

Appendix A

Since the data reported in this paper identify individuals as patients, in order to reflect the course of action associated with air pollution as well as the presence of other climate indicators, we use the average pollution indicator for the period prior to the patient's visit as a proxy for the degree of individual exposure to air pollution. The time window for air pollution is chosen to primarily reflect the temporal extent of the effect of air pollution prior to the patient's visit. Chang et al. (2018) focused on the effect of air pollution on insurance purchases using a 2-day window, while Deschênes et al. (2020) focused on the relationship between air pollution and obesity with a 12-month window.

Considering that the thermal inversions used as instrumental variables are daily variables, a corresponding moving average is also required. At shorter window lengths, the thermal inversions are exogenous to cost and visit duration, but because there is significant seasonal variation accompanied by thermal inversions, a longer window would make the instrumental variable seasonal and thus affect the exogeneity of the cost and visit duration. Therefore, the time window should not be too long. We checked the regression coefficient between 2 days and 15 days according to the settings in the literature (Tian et al., 2018; Liu et al., 2019; Wang et al., 2019).

The coefficients of β_1 in Equation (12) for the different time windows are shown in Figure a1. There was a significant effect of $PM_{2.5}$ on cost and visit duration, indicating low sensitivity of the effect of $PM_{2.5}$ with respect to the time window's significance; however, the overall coefficient becomes larger with longer windows, reflecting the cumulative process of the effect of air pollution.

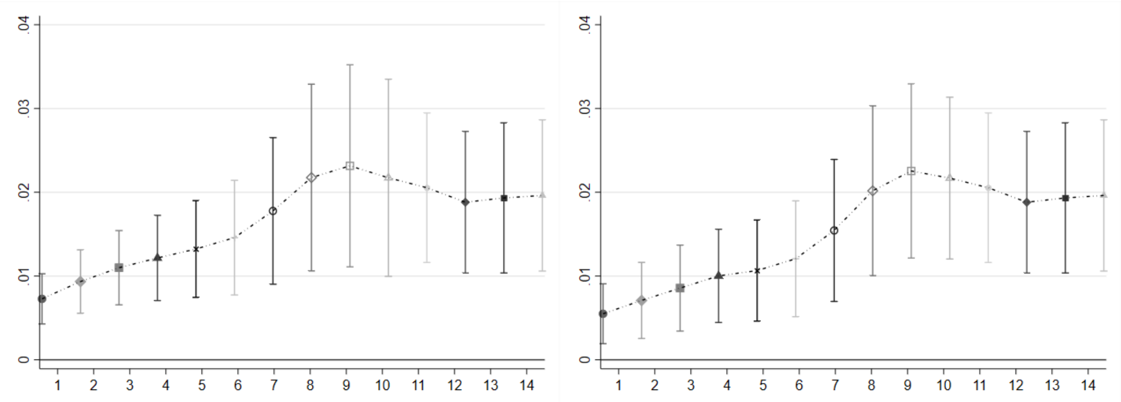

Figure 1a The lag effect of $PM_{2.5}$ on respiratory and circulatory patients' medical expenditure
Note. The horizontal axis shows the different time windows, and the vertical axis shows the regression coefficients.

Appendix B

First, the atmospheric temperature of the 925 hPa layer recorded using remote sensing satellites is obtained from the NASA-MERRA2[1] database. The raw NASA data has a resolution of 0.625°× 0.5°, which we interpolate to 0.05°× 0.05° using the local interpolation method. The atmospheric temperature recorded in 6-hour increments is averaged over the daily degree to obtain the temperature of the upper layers, denoted as $Temp_{ti}^{highlevel}$. Second, because the low-level atmospheric temperature is missing from the NASA database, we match the surface temperature data corresponding to the latitude and longitude. The surface temperature data are taken from surface monitoring stations to match the upper atmospheric temperature and interpolated to the same 0.05° × 0.05° resolution using the inverse distance weighted (IDW) method. This is denoted as $Temp_{ti}^{surface}$. When $Temp_{ti}^{highlevel} > Temp_{ti}^{surface}$, thermal inversions occur. The difference is the inverse temperature intensity. Otherwise, thermal inversions do not occur, and the corresponding thermal inversion intensity is recorded as 0.

After performing data extraction and interpolation as described above, the thermal inversion intensity of all the raster data within the sample city is arithmetically averaged on a daily basis to obtain the thermal inversion intensity for the corresponding date. Inversion intensity contains richer information and better reflects the actual inversion phenomenon (Sager, 2019).

Figure b1 shows the trend fit of the normalised daily $PM_{2.5}$ and inversion intensity. Both trends generally fit each other over time.

[1] Source: https://disc.gsfc.nasa.gov/datasets/M2I6NPANA_5.12.4/summary. Global Modeling and Assimilation Office (GMAO) (2015), MERRA-2 inst6_3d_ana_Np: 3d,6-Hourly,Instantaneous,Pressure-Level,Analysis,Analyzed Meteorological Fields V5.12.4, Greenbelt, MD, USA, Goddard Earth Sciences Data and Information Services Center (GES DISC), Accessed: [2020-10-21], 10.5067/A7S6XP56VZWS

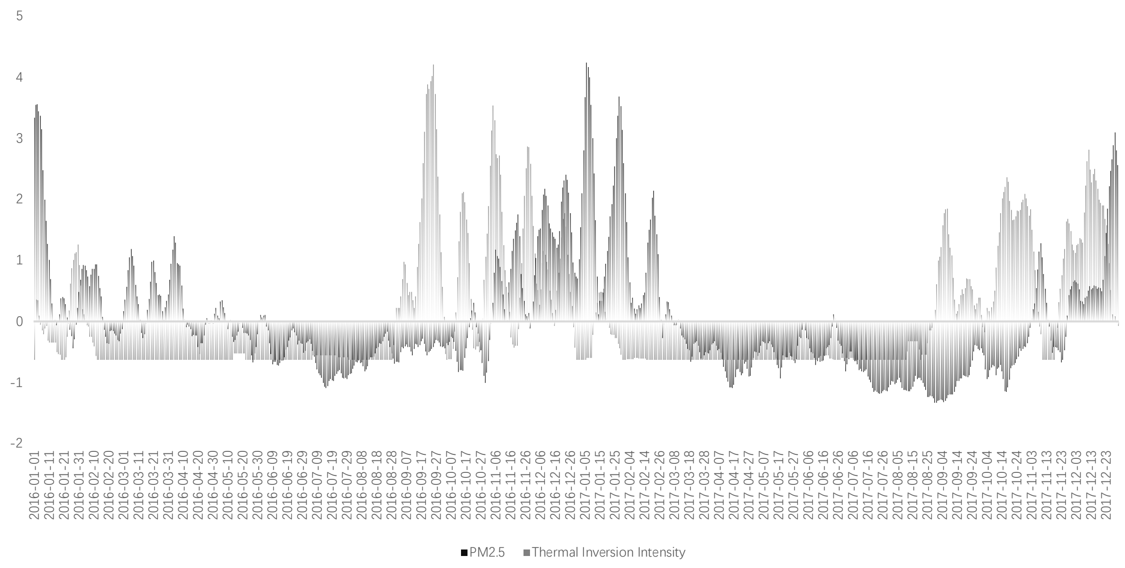

Figure b1 Temporal trends in $PM_{2.5}$ and thermal inversion intensity

Data sources: NASA-MERRA2 and data calculated by the authors

Appendix C

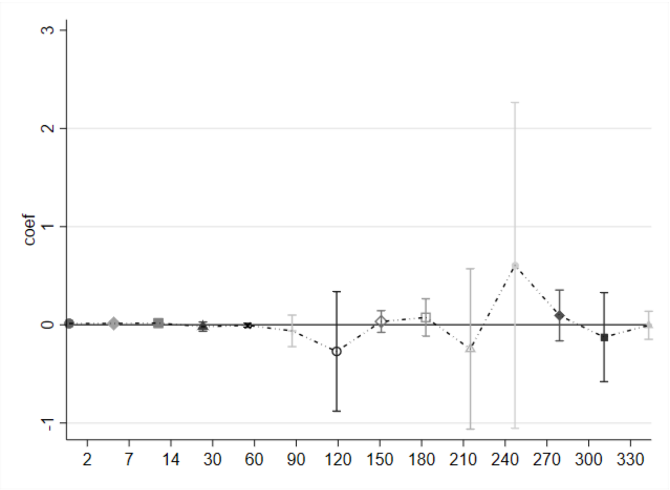

Figure c1 Impact of $PM_{2.5}$ on the cost of arthritis visits for different time windows

Reference

[1]Chang, T. Y., W. Huang, and Y. Wang. Something in the air: Pollution and the demand for health insurance[J]. Review of Economic Studies, 2018, 85(3): 1609–1634.

[2]Deschenes, O., H. Wang, S. Wang et al. The effect of air pollution on body weight and obesity: Evidence from China[J/OL]. Journal of Development Economics, 2020, 145(September 2019): 102461.

[3]Tian, Y., H. Liu, T. Liang et al. Ambient air pollution and daily hospital admissions: A nationwide study in 218 Chinese cities[J/OL]. Environmental Pollution, 2018, 242: 1042–

1049.
[4]Wang, C., L. Feng, and K. Chen. The impact of ambient particulate matter on hospital outpatient visits for respiratory and circulatory system disease in an urban Chinese population[J/OL]. Science of the Total Environment, 2019, 666: 672–679.
[5]Liu, C., R. Chen, F. Sera et al. Ambient particulate air pollution and daily mortality in 652 cities[J]. New England Journal of Medicine, 2019, 381(8): 705–715.
[6]Sager, L. Estimating the effect of air pollution on road safety using atmospheric temperature inversions[J]. Journal of Environmental Economics and Management, 2019, 98(251): 102250.
